# Joint Angular Kinematics and Gross Motor Function in Typically Developing Healthy Children

**DOI:** 10.3390/children12030280

**Published:** 2025-02-25

**Authors:** Monday Omoniyi Moses, Ngozi Florence Onuegbu, Prince De-Gualle Deku, Mary Abena Nyarko, Lydia Boampong Owusu, Abigael Omowumi Emikpe, Emmanuel Babatunde John, Rahul Soangra, Abiboye Cheduko Yifieyeh, Nicholas Akinwale Titiloye

**Affiliations:** 1 Department of Physiotherapy and Sports Science, Faculty of Allied Health Sciences, College of Health Sciences, Kwame Nkrumah University of Science & Technology, Kumasi AK-039, Ghana; nfonuegbu@knust.edu.gh (N.F.O.); pddeku@knust.edu.gh (P.D.-G.D.); manyarko@st.knust.edu.gh (M.A.N.); 2 Department of Nursing, School of Nursing and Midwifery, College of Health Sciences, Kwame Nkrumah University of Science & Technology, Kumasi AK-039, Ghana; 3 Department of Midwifery, School of Nursing and Midwifery, College of Health Sciences, Kwame Nkrumah University of Science & Technology, Kumasi AK-039, Ghana; abigael.emikpe@knust.edu.gh; 4 Department of Physical Therapy, Crean College of Health and Behavioral Sciences, Chapman University in Irvine, Irvine, CA 92866, USA; ejohn@ycp.edu (E.B.J.); soangra@chapman.edu (R.S.); 5 Myers School of Nursing and Health Professions, York College of Pennsylvania, York, PA 17403, USA; 6 Fowler School of Engineering, Chapman University, Orange, CA 92866, USA; 7 Department of Surgery, School of Medicine and Dentistry, College of Health Sciences, Kwame Nkrumah University of Science & Technology, Kumasi AK-039, Ghana; abiboye.yifieyeh@knust.edu.gh; 8 Department of Pathology, School of Medicine and Dentistry, College of Health Sciences, Kwame Nkrumah University of Science & Technology, Kumasi AK-039, Ghana; nicholastitiloye.sms@knust.edu.gh

**Keywords:** range of motion, lying and rolling, crawling and kneeling, walking, running and jumping, anthropometric

## Abstract

Objective: The aim of this study was to establish the interactions between joint angular kinematics and gross motor function in typically developing healthy Ghanaian children. Methods: A descriptive cross-sectional study design was employed. A total of 150 (69 (46.0%), 3.25 ± 0.08-year-old boys and 81 (54.0%), 3.25 ± 0.06-year-old girls) 2–4-year-old children were recruited. Joint angular kinematic variables [left hip flexion (LHF), left hip extension (LHE), right hip flexion (RHF), left knee flexion (LKF), right hip extension (RHE), left knee extension (LKE), right knee flexion (RKF), left ankle dorsi-flexion (LADF), right knee extension (RKE), right ankle plantar flexion (RAPF), left ankle plantar flexion (LAPF), and right ankle dorsi-flexion (RADF)] and gross motor function (lying and rolling, sitting, crawling and kneeling, standing, and walking, running, and jumping) were measured with standard scales. Results: The correlations between lying and rolling vs. RHE (r = 0.221; *p*-value < 0.01), LKE (r = −0.267; *p*-value < 0.01), LAPF (r = 0.264; *p*-value < 0.01), and RADF (r = 0.240; *p*-value < 0.01); crawling and kneeling vs. LKE (r = 0.196; *p*-value < 0.05) and RADF (r = 0.188; *p*-value < 0.05); and walking, running, and jumping vs. LKE (r = −0.214; *p*-value < 0.01) and RADF (r = −0.207; *p*-value < 0.05) were significant. Conclusions: There was a negative correlation between joint angular kinematics and total gross motor function in this sampled population. Typically, developing healthy children should be exposed to a range of motion, flexibility, and active transportation programs for optimal active lifestyles and improvements in gross motor skills.

## 1. Introduction

Gross motor function involves movements that use large muscles, such as lying, rolling, sitting, crawling, kneeling, standing, walking, running, and jumping [1,2]. Large muscles such as the gluteus medius and gluteus maximus muscles are essential for walking and running, stabilizing the pelvis, and controlling femoral adduction and internal rotation, while diagnosed weakness in these muscles can lead to musculoskeletal disorders [3].

Studies have shown that the gluteus medius stabilizes the pelvis and controls femoral adduction and internal rotation during gait, while the upper gluteus maximus fibers act as a hip external rotator and abductor [4,5,6]. Recent studies affirmed that the control of body movements, such as sitting, crawling, standing, and walking, allows children to move (efficient flexibility and range of motion) around in their surrounding environment efficiently and confidently [7,8,9].

Delays in gross motor development may indicate neurological disorders, and early identification is essential for successful treatments [10,11]. Also, gross motor function has been reported as important for figuring out kinematic and kinetic changes in growing children who have been diagnosed with a disorder [11,12,13]. In children with diseases like cerebral palsy, deficits in gross motor function influence movement and inform therapies to improve their quality of life [14].

Joint angular kinematics (JAK) has been explained as the study of the motion of joints concerning their angular displacement, velocity, and acceleration, without considering the forces that cause their motion [15]. JAK is seen as a fundamental aspect of biomechanics, providing critical insights into human movement, injury prevention, rehabilitation, and sports performance [15,16]. Therefore, contemporary research on the relationship between joint angular kinematics and gross motor function has focused on children with pathological disorders [17,18,19], with limited attention given to typically developing healthy children. It has been established that typically developing healthy children are often characterized by optimal presentations of physical, mental, intellectual, social, and emotional attributes [20,21,22,23].

The World Health Organization opined that children aged one to four years old experience rapid physical, cognitive, and emotional development [24]. Studying children within these age groups provides valuable insights into their early motor skill acquisition, including walking, running, and coordination [25,26]; cognitive and language development, which are foundational for later learning [27]; and social and emotional behaviors, as children begin forming attachments and emotional regulation patterns [28].

To ensure efficient development of interest in activities that use large muscles in typically developing healthy children, understanding joint angular kinematics (range of motion and flexibility) is inevitable. Passive range of motion in the lower limbs is a critical parameter for the diagnosis and treatment of musculoskeletal and neurological problems in children [29,30].

Previous studies have reiterated that the fundamental movement phase of motor development focuses on mastering the mechanics of skills that prepare children for the specialized movement phase of motor development, like cardiovascular endurance, muscular strength, and flexibility [16,31,32]. Although the interaction between joint angular kinematics and gross motor function among children in developing countries is seldom examined, to date, and to the best of the knowledge of the authors, there are limited studies on Ghanaian children. Giving specific attention to typically developing healthy Ghanaian children is essential to establish normative data for joint motion in different age groups; assess movement efficiency and postural control; and provide reference values for clinical and rehabilitative applications [33,34,35,36]. Furthermore, factors such as anthropometric differences (limb length, body mass, and muscle distribution), cultural variations in motor skill acquisition (e.g., play patterns and environmental influences), and nutritional and health status, which can affect motor performance, necessitate specific research in this population. Therefore, the objective of this study was to establish the interactions between joint angular kinematics and gross motor function in typically developing healthy children.

## 2. Materials and Methods

### 2.1. Study Design

This descriptive cross-sectional design study is part of comprehensive research on the sensor gait assessment and clinical gait parameters of typically developing Ghanaian children. The descriptive cross-sectional design uses data from participants at one moment to represent the population under investigation. The cross-sectional approach is particularly advantageous for assessing the prevalence of a phenomenon, characteristics of a population, or associations between variables within a defined timeframe [24]. This design facilitates the collection of data from a representative sample, allowing for broad generalizability while maintaining efficiency in terms of time and resources [25].

Moreover, as a subset of a broader research project, this cross-sectional study serves as a foundational component in generating baseline data that may inform further longitudinal or experimental inquiries [26]. Given its descriptive nature, this design does not establish causality but is instrumental in identifying patterns, trends, and potential relationships between key variables [27].

To ensure the children were representative of the population, random sampling was utilized. To analyze the descriptive cross-sectional research data obtained, frequency distributions, means, and standard deviations were used [28,29,30,31,32,33,34,35,36].

### 2.2. Participants

The sampled population was non-probabilistic, chosen for convenience, and subject to the parents’ acceptance to release their children to participate in this study. For the validity of this study, the sample size was calculated based on repeated measures, with factors having a medium effect size (Cohen’s f) of 0.3 and a power of 0.95, using G*Power 3.1.9.4. A total sample of 134 participants was required, with an additional 14.9% attrition rate (n = 20) included. However, a total of 150 (69 (46.0%), 3.25 ± 0.08-year-old boys and 81 (54.0%), 3.25 ± 0.06-year-old girls) from pre-primary schools fulfilled all the inclusion criteria and participated in this study.

The inclusion criteria were as follows: participants between the ages of 0 and 4, not living with any disability, and free from any diagnosed acute or chronic illnesses, including cardiovascular diseases, diabetes, asthma, and epilepsy, were included. Those with prenatal, perinatal, or postnatal complications and congenital birth defects were excluded. An introductory letter of intent with the objectives of this study was sent to more than fifty schools in Kumasi, the Ashanti region. After six months, few schools showed interest. All participants between the ages of 2 and 4 years were relatively healthy children who were assumed to be the eyes of the family very early in life and would be able to assist family members in household activities and taking care of each other [37] and had no reported history of neurological or motor impairments. The protocol was approved by the Institutional Ethics Committee (our ref.: CHRPE/AP/398/23). All participants, through class teachers, school heads, and parents, signed written informed consent in accordance with the Declaration of Helsinki.

### 2.3. Procedures and Instruments

Height and weight were measured with a WB-3000 digital stadiometer (Tanita, Tokyo, Japan), and the waist-to-hip ratio was calculated based on the circumferences of the waist and hip obtained through a non-elastic tape measure. Body mass index (BMI) was measured and recorded using a Tanita body composition analyzer (RD-953 model). The validity of the instrument to display reliable body mass indexes in preschool children has been well documented [38,39,40,41]. The angular kinematic variables (passive range of motion of the joints in the lower limbs) were measured using a 360° goniometer (Sunnyguli brand, Wenzhou, China, with a UPC of 798008035758) and recorded. The gross motor level of each participating child was measured using the Gross Motor Function Measure (GMFM-66 & GMFM-88) User’s Manual [41]. The GMFM-88 is divided into five dimensions: lying and rolling (4 items), sitting (15 items), crawling and kneeling (10 items), standing (13 items), and walking, running, and jumping (24 items) [41,42,43]. Using the GMFM-88, the children were assessed based on five dimensions (lying and rolling; sitting; crawling and kneeling; standing; and walking, running, and jumping) [41,42,44,45]. Each dimension was scored on a scale ranging from 0 to 3 (0 = does not initiate; 1 = initiates; 2 = partially completes; and 3 = completes) and a score of “NT” for items that were not tested. The goal score percentage for each dimension as well as the total score percentage was computed and documented at the end of each assessment [41]. The scientific reliability and utilization of the GMFM-88 have attracted the attention of researchers, especially among children with pathological and musculoskeletal disorders [43,46,47].

### 2.4. Statistical Analysis

The data normality was examined using the Kolmogorov–Smirnov test before exploring the interaction between results between the angular kinematic and gross motor function parameters. The collected data were entered into Microsoft Excel and exported to IBM Statistical Package for Social Sciences (SPSS) version 27.0, Chicago, IL, USA, for analysis. Descriptive analysis was used to represent the data as means, standard deviations, frequencies, and percentiles (Table 1). Pearson’s moment correlation coefficient was analyzed to determine the interaction between the angular kinematic and gross motor function variables (Table 2), and independent t-tests were used to determine gender differences between the angular kinematic and gross motor function parameters (Table 3). The confidence level was set at 95%.

## 3. Results

The demographic characteristics of the participants are shown in Table 1. The mean age shows that the majority of the children were below three years, while fifty-four were female and in the <50th body fat percentile.

The results from Table 2 based on the intervariable PPMC correlation show that there was a significant intercorrelation between RKE and RKF (r = 0.338; *p* < 0.05), RADF and RHE (r = −0.227; *p* < 0.05), RADF and RKE (r = 0.188; *p* < 0.05), RAPF and RHE (r = 0.217; *p* < 0.05), RAPF and RADF (r = −0.322; *p* < 0.05), LHF and RHF (r = 0.721; *p* < 0.05), LHF and RHE (r = 0.03; *p* < 0.05), LHE and RHE (r = 0.749; *p* < 0.05), LHE and RKE (r = 0.351; *p* < 0.05), LHE and RADF (r = −0.198; *p* < 0.05), LHE and RAPF (r = 0.308; *p* < 0.05), LKF and RKF (r = 0.859; *p* < 0.05), LKF and RKE (r = 0.831; *p* < 0.05), LKE and RKF (r = 0.421; *p* < 0.05), LKE and RKE (r = −0.180; *p* < 0.05), GMFM total% and RKE (r = −0.184; *p* < 0.05), GMFM E% and LAPF (r = −0.207; *p* < 0.05), and GMFM E% and RAPF (r = −0.214; *p* < 0.05). There was a significant positive correlation between the percentages of all GMFM variables and the GMFM total (*p* < 0.05) at a 95% confidence interval. Table 3 reveals that there was no significant difference between males and females at a 95% confidence interval in all the variables measured except height.

## 4. Discussion

This study established the interactions between angular kinematics and gross motor function in typically developing healthy children. Based on the findings, 54.0% (Table 1) of the children had a less than 50th body fat percentile rank without a significant gender difference in BMI (Table 3). This finding highlights that the participants were not at cardiovascular risk based on their body fat percentile and BMI, irrespective of gender affiliation, as postulated in [48]. The body fat levels support the suggestion that BMI values may not differ substantially between boys and girls within this sample [49]. This consistency could also be used to inform or refine standards in pediatric growth charts and health screening tools [50,51]. Clinically, children with body fat below the 50th percentile may have a lower risk of certain metabolic diseases [48], given the assumption that higher body fat often increases the risk of diabetes, cardiovascular diseases, and other metabolic disorders [14].

Based on Table 2, the findings from the current study show that the correlation between joint angular kinematics and total gross motor function in healthy children in the sampled population was negatively oriented, although positive with subdivisions. The implications of the negative correlation could indicate that elevated or more intricate angular kinematic metrics (e.g., specific joint angles or movement patterns) may not directly correspond to enhanced overall gross motor function in this demographic [52,53,54]. This could also mean that increased or decreased flexibility may or may not be responsible for a better quality of running, walking, jogging, and jumping because greater flexibility can enhance joint mobility, allowing for a more extensive stride length in running and jumping, improve overall efficiency, and reduce the risk of injury [55]. Nonetheless, the positive association across subdivisions of function may suggest that some subsets of gross motor abilities (walking, running, and jumping) are truly improved by specific angular kinematic attributes [56,57]. It has been indicated that more flexible muscles and tendons can absorb impact forces more effectively, reducing stress on joints and potentially improving movement mechanics [30,58].

Specifically, the correlation analysis in this study revealed that left and right hip flexion correlated negatively, though not significantly, with gross motor functions. This could indicate the presence of muscle imbalances, compensations, and restricted mobility in other muscle groups or joints, as suggested in [59]. This finding indicates the importance of joint kinematic evaluation to adopt one of five distinctive gait forms or skills: walking, running, galloping, hopping, and skipping [34,35] and the development of fundamental motor skills [60] as well as stability in children.

Additionally, it could lead to compromised movements, potentially affecting tasks such as walking, running, and an active lifestyle [61,62]. Furthermore, the correlation between left and right hip extension and gross motor function was also non-significantly negative. This negative correlation in this subdivision could indicate atypical neuromuscular development [63], although these findings are among relatively healthy children without diagnosed developmental conditions. Since overall gross motor function may not improve with general angular kinematics, researchers believe clinicians could focus on specific motor skills (lying and rolling, sitting, crawling and kneeling, and walking, running, and jumping) that correlate positively with kinematic variables [43,64,65]. It has been suggested that this approach could refine interventions to enhance targeted functions rather than global gross motor abilities [9]. Biomechanical, neuromuscular, or compensatory mechanisms may influence gross motor function more than hip flexion alone. While hip flexion may play a role in movement patterns, its direct contribution to gross motor function is uncertain, and other kinematic or kinetic factors may be more influential [33,35,58].

Another correlation result obtained revealed that left knee flexion correlated negatively with lying and rolling, sitting, crawling and kneeling, and standing, as well as positively with walking, running, and jumping. This finding suggests that limitations in basic developmental motor activities, like lying, rolling, sitting, crawling, kneeling, and standing, are associated with decreased left knee flexion or a restricted range of motion in knee flexion [63,64,65]. However, there has been an argument suggesting that an increase in knee flexion correlates positively with more dynamic activities like walking, running, and jumping [66,67].

One other major finding of this study was the significant and negative correlations observed between left knee extension and lying and rolling, as well as walking, running, and jumping, followed by a significant positive correlation with crawling and kneeling. The negative correlations between knee extension and lying, rolling, walking, running, and jumping may be explained by functional interpretation [68,69], where reduced knee extension (or decreased ability to fully extend the knee) may negatively impact movements that involve upright, weight-bearing activities and dynamic motions like walking, running, and jumping [66,70,71]. Growing evidence suggests that knee extension is crucial for efficient movement mechanics and stability in children, and any limitation could hinder their ability to perform motor skills tasks smoothly and safely [72,73]. However, there was no significant correlation between right knee extension and gross motor function.

The current study revealed that right ankle dorsi-flexion correlated significantly with lying and rolling, crawling and kneeling, and walking, running, and jumping, while left ankle plantar flexion correlated significantly with lying and rolling. This pattern supports earlier findings, which suggest that different ankle movements, such as dorsi-flexion and plantar flexion are linked to various functional movements, like lying and rolling, crawling and kneeling, and walking, running, and jumping, at different developmental stages of motor activities in children [54,74,75].

Gender-wise, Table 3 shows that there was no significant difference between the variables measured in the sample population, as previously reported [76,77]. This suggests the children shared similar growth patterns with the socio-cultural environment because growth patterns, including height, weight, and muscle mass development, influence the ability of children to perform gross motor tasks, such as walking, running, jumping, and balance. Studies indicate that children with similar growth trajectories often have comparable neuromuscular coordination and postural control, leading to similar motor development milestones [78,79]. The WHO Multicentre Growth Reference Study [80] found that across different countries, children raised in similar socio-economic and nutritional conditions displayed similar motor milestone timelines. Malina and Bouchard [81] also demonstrated that children from similar ethnic and socio-cultural backgrounds had parallel motor skill progressions, even when raised in different geographic locations. The literature has shown that cultural differences in infant handling (e.g., swaddling vs. free movement) influence postural control and early gross motor milestones [82,83]. This result provides a benchmark for clinicians evaluating children with motor impairments and suggests that in healthy children, normal variations in joint angles or motion patterns (angular kinematics) do not significantly impact their gross motor function.

The limitations of this study include that it was carried out in non-clinical settings on relatively healthy children. Although the sample was a vulnerable population, the sample size for this study may not have been sufficient to be a very good representative population. However, the G*Power software was used to ensure the sample was at least sufficient for this study.

## 5. Conclusions

There was a negative correlation between joint angular kinematics and total gross motor function in this sampled population. Also, there were significant negative correlations between left knee extension and lying and rolling, as well as walking, running, and jumping, followed by significant positive correlations with crawling and kneeling. Furthermore, right ankle dorsi-flexion correlated significantly with lying and rolling, crawling and kneeling, and walking, running, and jumping, while left ankle plantar flexion correlated significantly with lying and rolling. It is recommended that typically developing healthy children should be exposed to a range of motion, flexibility, and active transportation programs for optimal active lifestyles and improvements in gross motor skills.

## Figures and Tables

**Table 1:** Mean and percentile distribution of outcome measures.

Variable	Mean ± Std. Deviation	Min.	Max.	Percentile		
				25th	50th (Median)	75th
Gender (N, %)	M (69,46.0); F (81, 54.0)					
Grouped body fat percentile (N, %)	<50th (81, 54.0); 50th–59th (10, 6.7); 60th–69th (16, 10.7); 70th–79th (9, 6.0); 80th–89th (10, 6.7); >90th (24, 16.0)					
Age group (year) (N, %)	2.0–2.99 (38, 25.3); 3.0–3.99 (90, 60.0); 4.0–4.99 (22, 14.7)					
Age (year)	3.25 ± 0.64	2.00	4.40	2.90	3.30	3.0
Height (cm)	99.26 ± 7.56	83.60	144.00	94.92	99.10	102.40
Weight (kg)	15.61 ± 2.75	10.3	27.7	13.77	15.45	17.10
BMI kg/m^2^	15.91 ± 2.09	9.3	25.7	14.50	15.60	16.60
WHR (cm)	0.93 ± 0.04	0.82	1.12	0.90	0.93	0.96
RHF (°)	112.91 ± 14.76	11	135	105.75	115.00	123.00
RHE (°)	26.82 ± 4.06	17.0	35.0	24.00	27.00	30.00
RKF (°)	125.26 ± 8.63	101	138	120.00	128.00	132.00
RKE (°)	119.45 ± 17.75	23	140	112.75	124.50	130.00
RADF (°)	20.75 ± 4.85	12.0	37.0	18.00	20.00	20.00
RAPF (°)	33.50 ± 8.71	14	50	26.75	34.00	40.00
LHF (°)	111.67 ± 11.76	79	134	105.00	111.50	120.00
LHE (°)	26.73 ± 4.58	19	39	23.00	27.00	29.00
LKF (°)	124.29 ± 9.32	90.0	139.4	119.00	127.00	130.00
LKE (°)	119.72 ± 16.17	25	138	113.75	123.00	130.00
LADF (°)	20.52 ± 4.83	12.0	36.0	18.00	19.00	22.00
LAPF (°)	33.25 ± 8.65	14	50	25.75	33.00	40.00
GMFM A%	77.25 ± 22.45	1.9	100.0	66.67	82.40	96.10
GMFM B%	89.18 ± 15.13	11.7	100.0	86.70	95.00	100.00
GMFM C%	91.04 ± 16.34	9.5	100.0	90.30	96.10	100.00
GMFM D%	90.82 ± 19.08	10.2	100.0	91.42	100.00	100.00
GMFM E%	84.22 ± 20.94	4.2	100.0	82.72	93.05	95.80
GMFM total%	84.33 ± 17.42	6.00	99.50	82.87	90.05	94.52

**Table 2:** Correlations comparison between angular kinematic and gross motor function variables.

Gross Motor Function	Lying and Rolling	Sitting	Crawling and Kneeling	Standing	Walking, Running, and Jumping	GMFM Total%
Angular kinematic variables	Pearson correlation	Pearson correlation	Pearson correlation	Pearson correlation	Pearson correlation	Pearson correlation
Left hip flexion	−0.105	−0.104	−0.017	−0.001	−0.049	−0.113
Right hip flexion	0.068	0.020	0.081	−0.008	−0.036	−0.005
Left hip extension	−0.035	−0.032	−0.015	0.010	−0.060	−0.087
Right hip extension	0.221 **	0.081	−0.090	−0.059	0.143	0.032
Left knee flexion	−0.050	−0.061	−0.010	−0.048	0.104	0.065
Right knee flexion	−0.158	−0.062	0.045	0.012	−0.057	−0.094
Left knee extension	−0.267 **	−0.141	0.196 *	−0.098	−0.214 **	−0.131
Right knee extension	0.027	−0.032	0.029	−0.030	−0.069	−0.055
Left ankle dorsi-flexion	−0.090	−0.059	−0.039	0.034	−0.040	−0.110
Right ankle dorsi-flexion	0.240 **	−0.142	0.188 *	−0.095	−0.207 *	−0.088
Right ankle plantar flexion	−0.048	−0.016	0.086	0.006	−0.044	0.018
Left ankle plantar flexion	0.264 **	0.115	−0.014	−0.052	0.150	0.054

** Correlation is significant at the 0.01 level (2-tailed), * correlation is significant at the 0.05 level (2-tailed), and % gross motor function measure = GMFM total%.

**Table 3:** Comparison between angular kinematic and gross motor function variables by gender.

Variable	Gender	Mean Rank	Mean ± Std.	Mean Diff.	T	*p*-Value	95% CI
Age (year)	M	76.36	3.25 ± 0.08	−0.0034	−0.033	0.974	−0.2131, 0.20623
	F	74.77	3.25 ± 0.06				
Height (cm)	M	82.78	100.85 ± 9.37	2.9251	2.396	0.018 *	0.51262, 5.33760
	F	69.30	97.92 ± 5.29				
Weight (kg)	M	77.44	15.66 ± 2.50	0.0888	0.196	0.845	−0.8058, 0.9834
	F	73.85	15.57 ± 2.96				
BMI (kg/m^2^)	M	70.51	15.74 ± 2.22	−0.3084	−0.899	0.370	−0.9863, 0.3696
	F	79.75	16.05 ± 1.97				
WHR (cm)	M	79.86	0.93 ± 0.04	0.0074	1.133	0.259	−0.00554, 0.02045
	F	71.79	0.92 ± 0.03				
RHF (°)	M	74.07	113.61 ± 10.81	1.288	0.531	0.596	−3.503, 6.079
	F	76.72	112.32 ± 17.48				
RHE (°)	M	76.77	26.90 ± 4.00	0.1484	0.222	0.824	−1.1710, 1.4678
	F	74.42	26.75 ± 4.13				
RKF (°)	M	72.23	124.67 ± 8.67	−1.099	−0.775	0.439	−3.899, 1.702
	F	78.28	125.77 ± 8.63				
RKE (°)	M	75.36	119.49 ± 18.60	0.085	0.029	0.977	−5.683, 5.853
	F	75.62	119.41 ± 17.11				
RADF (°)	M	77.28	20.93 ± 4.75	0.3436	0.431	0.667	−1.2325, 1.9198
	F	73.99	20.59 ± 4.96				
RAPF (°)	M	70.14	32.51 ± 8.45	−1.838	−1.290	0.199	−4.655, 0.978
	F	80.07	34.35 ± 8.90				
LHF (°)	M	72.59	111.35 ± 10.60	−0.590	−0.305	0.760	−4.411, 3.230
	F	77.98	111.94 ± 12.72				
LHE (°)	M	72.97	26.33 ± 4.18	−0.741	−0.987	0.325	−2.225, 0.743
	F	77.65	27.07 ± 4.89				
LKF (°)	M	74.75	124.23 ± 9.15	−0.1080	−0.070	0.944	−3.1371, 2.9211
	F	76.14	124.34 ± 9.52				
LKE (°)	M	79.20	121.67 ± 14.32	3.605	1.364	0.175	−1.617, 8.826
	F	72.35	118.06 ± 17.51				
LADF (°)	M	75.19	20.78 ± 4.81	0.4777	0.602	0.548	−1.0906, 2.0460
	F	75.77	20.30 ± 4.86				
LAPF (°)	M	71.61	32.43 ± 8.26	−1.503	−1.061	0.290	−4.303, 1.296
	F	78.81	33.94 ± 8.95				
GMFM A%	M	72.97	76.86 ± 21.58	−0.7239	−0.196	0.845	−8.0158, 6.5680
	F	77.65	77.58 ± 23.29				
GMFM B%	M	74.41	90.77 ± 10.70	2.9379	1.187	0.237	−1.9543, 7.8301
	F	76.43	87.83 ± 18.03				
GMFM C%	M	74.33	89.63 ± 19.19	−2.6049	−0.973	0.332	−7.8982, 2.6883
	F	76.49	92.23 ± 13.46				
GMFM D%	M	75.09	90.40 ± 19.75	−0.7736	−0.247	0.806	−6.9722, 5.4249
	F	75.85	91.17 ± 18.61				
GMFM E%	M	76.69	85.01 ± 21.11	1.4744	0.429	0.669	−5.3246, 8.2734
	F	74.49	83.54 ± 20.90				
GMFM total%	M	75.79	84.42 ± 17.31	0.16772	0.059	0.953	−5.49372, 5.82917
	F	75.25	84.25 ± 17.62				

* Significant difference at *p* < 0.05.

## Data Availability

Data will be made available upon request. The data presented in this study are available on request from the corresponding author due to privacy, legal and ethical reasons.

## Abbreviations

The following abbreviations were used in this manuscript:

BMI	Body mass index
LHF (°)	Left hip flexion
WHR-	Waist-to-hip ratio
LHE (°)	Left hip extension
RHF (°)	Right hip flexion
LKF (°)	Left knee flexion
RHE (°)	Right hip extension
LKE (°)	Left knee extension
RKF (°)	Right knee flexion
LADF (°)	Left ankle dorsi-flexion
RKE (°)	Right knee extension
RAPF (°)	Right ankle plantar flexion
LAPF (°)	Left ankle plantar flexion
RADF (°)	Right ankle dorsi-flexion
GMFM A%	(Lying and rolling) total dimension in A ÷ 51 × 100
GMFM B%	(Sitting) total dimension in B ÷ 60 × 100
GMFM C%	(Crawling and kneeling) total dimensions in c ÷ 42 × 100
GMFM D%	(Standing) total dimensions in D ÷ 39 × 100
GMFM E%	(Walking, running, and jumping) total dimensions in E ÷ 72 × 100
GMFM total%	(A% + B% + C% + D% + E%) ÷ 5

## References

[1] Brito-Suárez J.M., Camacho-Juárez F., Sánchez-Medina C.M., Hernández-Pliego G., Gutiérrez-Camacho C. (2022). Gross motor disorders in pediatric patients with acute lymphoblastic leukemia and survivors: A systematic review. Pediatr. Hematol. Oncol..

[2] Abd El-Hady S.S., Abd El-Azim F.H., El-Talawy H.A.E.A.M. (2018). Correlation between cognitive function, gross motor skills and health—Related quality of life in children with Down syndrome. Egypt. J. Med. Hum. Genet..

[3] Baik S.-M., Cynn H.-S., Shim J.-H., Lee J.-H., Shin A.-R., Lee K.-E. (2021). Effects of Log-Rolling Position on Hip-Abductor Muscle Activation During Side-Lying Hip-Abduction Exercise in Participants with Gluteus Medius Weakness. J. Athl. Train..

[4] Hammond K.E., Kneer L., Cicinelli P. (2021). Rehabilitation of Soft Tissue Injuries of the Hip and Pelvis. Clin. Sports Med..

[5] Sirico F., Palermi S., Massa B., Corrado B. (2020). Tendinopathies of the hip and pelvis in athletes: A narrative review. J. Hum. Sports Exerc..

[6] Parcells B.W. (2020). Pediatric Hip and Pelvis. Pediatr. Clin. N. Am..

[7] Jinhua L., Lijin Z. (2024). Motor Development. The ECPH Encyclopedia of Psychology [Internet].

[8] Cuesta-Gómez A., Fernández-González P., Carratalá-Tejada M., Aguilar-Bejines I. (2024). Differences in Motor Development between Preterm Infants and Full-Term Preschool Children. Children.

[9] Seamon B.A., Sears C.L., Anderson E., Velozo C.A. (2024). Defining a universal measurement unit and scale for gross motor development. Front. Rehabil. Sci..

[10] Hadders-Algra M. (2021). Early diagnostics and early intervention in neurodevelopmental disorders—Age-dependent challenges and opportunities. J. Clin. Med..

[11] Walsh K. (2010). The Investigation of Social Competency as Related to Gross Motor Skills in Children with Developmental Delays [Internet]. [Canada]: McGill University. https://escholarship.mcgill.ca/concern/theses/xw42n824x.

[12] Schwartz M.H., Rozumalski A., Trost J.P. (2008). The effect of walking speed on the gait of typically developing children. J. Biomech..

[13] Cardoso M.R., Armstrong D.P., Fischer S.L., Albert W.J. (2023). Differential effects of sex on upper body kinematics and kinetics during fatiguing, Asymmetric lifting. Appl. Ergon..

[14] Jackman M., Sakzewski L., Morgan C., Boyd R.N., Brennan S.E., Langdon K., Toovey R.A.M., Greaves S., Thorley M., Novak I. (2021). Interventions to improve physical function for children and young people with cerebral palsy: International clinical practice guideline. Dev. Med. Child Neurol..

[15] Winter D.A. (2009). Biomechanics and Motor Control of Human Movement.

[16] Thamm A. (2021). Improving Gait in Cerebral Palsy Effects of a Combined Strength, Flexibility and Gait Training Intervention on Lower Limb Gait Kinematics and Kinetics in Children and Young Adults with Spastic Cerebral Palsy. Master’s Thesis in Biomechnics.

[17] Louey M.G.Y., Harvey A., Passmore E., Grayden D., Sangeux M. (2024). Kinematic upper limb analysis outperforms electromyography at grading the severity of dystonia in children with cerebral palsy. Clin. Biomech..

[18] Homayounpour M., Gomez N.G., Vasavada A.N., Merryweather A.S. (2021). The role of neck muscle co-contraction and postural changes in head kinematics after safe head impacts: Investigation of head/neck injury reduction. J. Biomech..

[19] Hajihosseinali M., Behzadipour S., Taghizadeh G., Farahmand F. (2022). Direction-dependency of the kinematic indices in upper extremities motor assessment of stroke patients. Med. Eng. Phys..

[20] Park M., Budisavljević S., Alemán-Díaz A.Y., Carai S., Schwarz K., Kuttumuratova A., Jobe L.B., Hülsen V., Lee Y.E., Scott E. (2023). Child and adolescent health in Europe: Towards meeting the 2030 agenda. J. Glob. Health.

[21] Alhusaini A.A. (2021). A Review of the Literature on Physical Activity, Screen Time, and Sedentary Behavior of Typically Developing Healthy Children in Saudi Arabia. Crit. Rev. Phys. Rehabil. Med..

[22] Perrin J.M., Lu M.C., Geller A., DeVoe J.E. (2019). Vibrant and Healthy Kids: Aligning Science, Practice, and Policy to Advance Health Equity. Acad. Pediatr..

[23] Cebotari V., Mazzucato V., Siegel M. (2016). Child Development and Migrant Transnationalism: The Health of Children Who Stay Behind in Ghana and Nigeria. J. Dev. Stud..

[24] De Onis M., Onyango A.W., Borghi E., Siyam A., Nishida C., Siekmann J. (2007). Development of a WHO growth reference for school-aged children and adolescents. Bull. World Health Organ..

[25] Adolph K.E., Hoch J.E. (2019). Motor development: Embodied, embedded, enculturated, and enabling. Annu. Rev. Psychol..

[26] Thelen E., Turkewitz G., Devenny D.A. (2013). Timing and developmental dynamics in the acquisition of early motor skills. Developmental Time and Timing.

[27] Hoff E. (2013). Interpreting the early language trajectories of children from low-SES and language minority homes: Implications for closing achievement gaps. Dev. Psychol..

[28] Thompson R.A., Cassidy J., Shaver P.R. (2016). Early attachment and later development: Reframing the questions. Handbook of Attachment: Theory, Research and Clinical Applications.

[29] Wu Y.-N., Hwang M., Ren Y., Gaebler-Spira D., Zhang L.-Q. (2011). Combined passive stretching and active movement rehabilitation of lower-limb impairments in children with cerebral palsy using a portable robot. Neurorehabilit. Neural Repair.

[30] Whitall J., Savelsbergh G., Davids K., Van der Kamp J., Bennett S.J. (2013). Development of locomotor co-ordination and control in children. Development of Movement Coordination in Children: Applications in the Field of Ergonomics, Health Sciences and Sport.

[31] Reeves L., Broeder C.E., Kennedy-Honeycutt L., Matney L. (1999). Relationship of Fitness and Gross Motor Skills For Five- to Six-Yr.-Old Children. Percept. Orz. Mofor Ski..

[32] Valadão P., Piitulainen H., Haapala E.A., Parviainen T., Avela J., Finni T. (2021). Exercise intervention protocol in children and young adults with cerebral palsy: The effects of strength, flexibility and gait training on physical performance, neuromuscular mechanisms and cardiometabolic risk factors (EXECP). BMC Sports Sci. Med. Rehabil..

[33] Ito T., Ito Y., Nakai A., Sugiura H., Noritake K., Kidokoro H., Natsume J., Ochi N. (2021). Bilateral asymmetry in the gait deviation index in school-aged children with the trait of developmental coordination disorder. Gait Posture.

[34] Armand S., Decoulon G., Bonnefoy-Mazure A. (2016). Gait analysis in children with cerebral palsy. EFORT Open Rev..

[35] Baker R., McGinley J.L., Schwartz M.H., Beynon S., Rozumalski A., Graham H.K., Tirosh O. (2009). The Gait Profile Score and Movement Analysis Profile. Gait Posture.

[36] Schwartz M.H., Rozumalski A. (2008). The gait deviation index: A new comprehensive index of gait pathology. Gait Posture.

[37] Ettinger A.S. (2004). Children’s Health, the Nation’s Wealth: Assessing and Improving Child Health. Environ. Health Perspect..

[38] Louie L., Chan L. (2003). The use of pedometry to evaluate the physical activity levels among preschool children in Hong Kong. Early Child Dev. Care.

[39] Nyström C.D., Henriksson P., Alexandrou C., Löf M. (2016). The Tanita SC-240 to assess body composition in pre-school children: An evaluation against the three component model. Nutrients.

[40] Gutiérrez-Marín D., Escribano J., Closa-Monasterolo R., Ferré N., Venables M., Singh P., Wells J.C., Muñoz-Hernando J., Zaragoza-Jordana M., Gispert-Llauradó M. (2021). Validation of bioelectrical impedance analysis for body composition assessment in children with obesity aged 8-14y. Clin. Nutr..

[41] Russell D.J., Rosenbaum P., Wright M., Avery L.M. (2021). Gross Motor Function Measure (GMFM-66 & GMFM-88) Users Manual.

[42] Te Velde A., Morgan C. (2022). Gross Motor Function Measure (GMFM-66 & GMFM-88) User’s Manual, 3rd ed. Book Review. Pediatr. Phys. Ther..

[43] Salavati M., Rameckers E., Waninge A., Krijnen W., Steenbergen B., van der Schans C. (2017). Gross motor function in children with spastic Cerebral Palsy and Cerebral Visual Impairment: A comparison between outcomes of the original and the Cerebral Visual Impairment adapted Gross Motor Function Measure-88 (GMFM-88-CVI). Res. Dev. Disabil..

[44] Mongold S.J., Georgiev C., Legrand T., Bourguignon M. (2023). Afferents to Action: Cortical Proprioceptive Processing Assessed with Corticokinematic Coherence Specifically Relates to Gross Motor Skills. Eneuro.

[45] Palisano R.J., Rosenbaum P., Bartlett D., Livingston M.H. (2008). Content validity of the expanded and revised Gross Motor Function Classification System. Dev. Med. Child Neurol..

[46] Gavazzi F., Patel V., Charsar B., Glanzman A., Erler J., Sevagamoorthy A., McKenzie E., Kornafel T., Ballance E., Pierce S.R. (2023). Gross Motor Function in Pediatric Onset *TUBB4A*-Related Leukodystrophy: GMFM-88 Performance and Validation of GMFC-MLD in TUBB4A. J. Child Neurol..

[47] CanChild (2022). The GMFM-88 and GMFM-Institute for Applied Health Sciences.

[48] Chung S.T., Onuzuruike A.U., Magge S.N. (2018). Cardiometabolic Risk in Obese Children.

[49] Ma F.-F., Luo D.-M. (2023). Relationships between physical activity, fundamental motor skills, and body mass index in preschool children. Front. Public Health.

[50] Ozturk A., Tartar A., Huseyinsinoglu B.E., Ertas A.H. (2016). A clinically feasible kinematic assessment method of upper extremity motor function impairment after stroke. Measurement.

[51] Guimarães E., Baxter-Jones A., Maia J., Fonseca P., Santos A., Santos E., Tavares F., Janeira M.A. (2019). The Roles of Growth, Maturation, Physical Fitness, and Technical Skills on Selection for a Portuguese Under-14 Years Basketball Team. Sports.

[52] Bechet R., Tisserand R., Fradet L., Colloud F. (2024). Evidence of invariant lower-limb kinematics in anticipation of ground contact during drop-landing and drop-jumping. Hum. Mov. Sci..

[53] Carcreff L., Moulin C., Mariani B., Armand S. (2022). Simple rule to automatically recognize the orientation of the sagittal plane foot angular velocity for gait analysis using IMUs on the feet of individuals with heterogeneous motor disabilities. J. Biomech..

[54] Ugbolue U.C., Robson C., Donald E., Speirs K.L., Dutheil F., Baker J.S., Dias T., Gu Y. (2021). Joint Angle, Range of Motion, Force, and Moment Assessment: Responses of the Lower Limb to Ankle Plantarflexion and Dorsiflexion. Appl. Bionics Biomech..

[55] Behm D.G., Chaouachi A. (2011). A review of the acute effects of static and dynamic stretching on performance. Eur. J. Appl. Physiol..

[56] Piscitelli D., Ferrarello F., Ugolini A., Verola S., Pellicciari L. (2021). Measurement properties of the Gross Motor Function Classification System, Gross Motor Function Classification System-Expanded & Revised, Manual Ability Classification System, and Communication Function Classification System in cerebral palsy: A systematic review with meta-analysis. Dev. Med. Child Neurol..

[57] Scherff E., Schnell S.E., Siebert T., D’Souza S. (2024). Reference measures of lower-limb joint range of motion, muscle strength, and selective voluntary motor control of typically developing children aged 5–17 years. J. Child. Orthop..

[58] Magnusson P., Renström P. (2006). The European College of Sports Sciences position statement: The role of stretching exercises in sports. Eur. J. Sport Sci..

[59] Comerford M.J., Mottram S.L. (2001). Review article: Movement and stability dysfunction-Contemporary developments. Man. Ther..

[60] Savelsbergh G., Davids K., van der Kamp J., Bennett S.J. (2013). Development of Movement Co-Ordination in Children: Applications in the Fields of Ergonomics, Health Sciences and Sport.

[61] Chen C., Yang S., Tang Y., Yu X., Chen C., Zhang C., Luo F. (2023). Correlation between strength/endurance of paraspinal muscles and sagittal parameters in patients with degenerative spinal deformity. BMC Musculoskelet. Disord..

[62] Han G., Zhou S., Qiu W., Fan Z., Yue L., Li W., Li W. (2023). Role of the Paraspinal Muscles in the Sagittal Imbalance Cascade: The Effects of Their Endurance and of Their Morphology on Sagittal Spinopelvic Alignment. J. Bone Jt. Surg..

[63] Manikowska F., Brazevič S., Jóźwiak M., Lebiedowska M.K. (2022). Contribution of Different Impairments to Restricted Knee Flexion during Gait in Individuals with Cerebral Palsy. J. Pers. Med..

[64] Marsico P., Tal-Akabi A., van Hedel H.J.A. (2016). The relevance of nerve mobility on function and activity in children with Cerebral Palsy. BMC Neurol..

[65] MacWilliams B.A., Prasad S., Shuckra A.L., Schwartz M.H. (2022). Causal factors affecting gross motor function in children diagnosed with cerebral palsy. PLoS ONE.

[66] Souza R.B., Fang C., Luke A., Wu S., Li X., Majumdar S. (2012). Relationship between knee kinetics during jumping tasks and knee articular cartilage MRI T1rho and T2 relaxation times. Clin. Biomech..

[67] Đorđić V., Tubić T., Jakšić D. (2016). The Relationship between Physical, Motor, and Intellectual Development of Preschool Children. Procedia-Soc. Behav. Sci..

[68] Ansa O.E.O., Mprah K.W., Moses M.O., Owusu I., Acheampong E. (2021). Effect of Community-Based Functional Aerobic Training on Motor Performance and Quality of Life of Children with Spastic Cerebral Palsy. Ethiop. J. Health Sci..

[69] Labaf S., Shamsoddini A., Hollisaz M.T., Sobhani V., Shakibaee A. (2015). Effects of Neurodevelopmental Therapy on Gross Motor Function in Children with Cerebral Palsy. Iran J. Child. Neurol..

[70] Pierce S.R., Skorup J., Paremski A.C., Prosser L.A. (2021). The relationship between the Family Empowerment Scale and Gross Motor Function Measure-66 in Young Children with cerebral palsy. Child. Care Health Dev..

[71] Zeng N., Ayyub M., Sun H., Wen X., Xiang P., Gao Z. (2017). Effects of physical activity on motor skills and cognitive development in early childhood: A systematic review. BioMed Res. Int..

[72] Lee D., Shepherd M.K., Mulrine S.C., Schneider J.D., Moore K.F., Eggebrecht E.M., Rogozinski B.M., Herrin K.R., Young A.J. (2023). Reducing Knee Hyperextension With an Exoskeleton in Children and Adolescents with Genu Recurvatum: A Feasibility Study. IEEE Trans. Biomed. Eng..

[73] Nyland J., Pyle B., Richards J., Yoshida K., Brey J., Carter S. (2023). A clinical practice review of therapeutic movement-based anterior cruciate ligament reconstruction return to sports bridge program: The biological, biomechanical and behavioral rationale. Ann. Jt..

[74] Fox E.J., Moon H., Kwon M., Chen Y.-T., Christou E.A. (2014). Neuromuscular control of goal-directed ankle movements differs for healthy children and adults. Eur. J. Appl. Physiol..

[75] Petersen T.H., Kliim-Due M., Farmer S.F., Nielsen J.B. (2010). Childhood development of common drive to a human leg muscle during ankle dorsiflexion and gait. J. Physiol..

[76] Anieto E.M., Anieto I.B., Ituen O.A., Naidoo N., Ezema C.I., Smits-Engelsman B. (2023). The relationship between kinaesthesia, motor performance, physical fitness and joint mobility in children living in Nigeria. BMC Pediatr..

[77] Raudsepp L., Pääsuke M. (1995). Gender Differences in Fundamental Movement Patterns, Motor Performances, and Strength Measurements of Prepubertal Children. Pediatr. Exerc. Sci..

[78] Cech D.J., Martin S.T. (2023). Functional Movement Development Across the Life Span.

[79] Rao P., Vander Schaaf E.B., Steiner M.J., Perry M., Halpern-Felsher B. (2023). Normal child growth and development. Encyclopedia of Child and Adolescent Health.

[80] de Onis M., Garza C., Victora C.G., Onyango A.W., Frongillo E.A., Martines J. (2004). The WHO Multicentre Growth Reference Study: Planning, study design, and methodology. Food Nutr. Bull..

[81] Malina R., Bouchard C., Bar-Or O. (2004). Growth, Maturation, and Physical Activity.

[82] Hadders-Algra M. (2005). Development of postural control during the first 18 months of life. Neural Plast..

[83] Hadders-Algra M. (2018). Early human motor development: From variation to the ability to vary and adapt. Neurosci. Biobehav. Rev..

